# Mental Health literacy: Perspectives from Northern Kenya Turkana adolescents

**DOI:** 10.1192/j.eurpsy.2023.1022

**Published:** 2023-07-19

**Authors:** P. Wadende, T. Sodi

**Affiliations:** 1 Education and Human Resource development, Kisii University, Kenya, Kericho, Kenya; 2 Psychology, University of Limpopo, Limpopo, South Africa

## Abstract

**Introduction:**

Mental illness accounts for high levels of morbidity, mortality, and poor quality of life among young people. Globally, 1 in 7 youth between 10-19 years are mentally ill making up 13% of the global burden of disease; depression, anxiety, conduct disorders, and attention-deficit/hyperactivity disorder lead here. Unaddressed mental illness progresses into adulthood disrupting victims’ lives. In Kenya, school going adolescent mental illness is manifested in violent outbursts such as arson attacks, intimate partner violence and riots in school. Not much is documented of non-school going adolescents and children in Kenya even while it is estimated that they number about 1.8 million. To seek help for mental illness, one has to recognize and understand it causes.

**Objectives:**

An ethnographic study exploring rural based Kenyan adolescents’ conceptualizations, causes and management options for Depression, Schizophrenia and anxiety among the rural Turkana community of Northern Kenya.

**Methods:**

An ethnographic study exploring rural based Kenyan adolescents’ conceptualizations, causes and management options for Depression, Schizophrenia and anxiety among the rural Turkana community of Northern Kenya. We conducted Focus Group Discussions with 32 adolescents between the ages of 13 and 17 divided into four groups; school going girls and non-school going girls and a similar two groups for boys. We first read out scenarios in which the main character exhibited symptoms of one of the three mental health conditions and analyzed the data thematically.

**Results:**

32 participants described the health conditions without referring to the local names we had collected earlier; Depression (*Akiyalolong*) Schizophrenia (*waarit/ Ngikerep*) Anxiety (*Ngatameta naaronok*). Participants conceived the three conditions as resulting from extreme sadness attending loss, traumatic event, curses and rarely as mental illness. They assigned curses, guilt, hunger pangs, evil spells as causes and believed friends and age-mates, parents, teachers, the local chief among other options could help and rarely medical intervention.

**Conclusions:**

Interventions to improve the adolescent’s knowledge of mental illness is needed so they can seek help for themselves and possibly help others.

**Disclosure of Interest:**

None Declared

